# Remote Technologies and Filial Obligations at a Distance: New Opportunities and Ethical Challenges

**DOI:** 10.1007/s41649-023-00256-3

**Published:** 2023-09-01

**Authors:** Yi Jiao (Angelina) Tian, Fabrice Jotterand, Tenzin Wangmo

**Affiliations:** 1 Institute for Biomedical Ethics, University of Basel, Basel, Switzerland; 2 Center for Bioethics and Medical Humanities, Medical College of Wisconsin, Milwaukee, USA

**Keywords:** Remote monitoring technologies, Filial obligations, Distance caregiving, Well-being of old age, Age in place

## Abstract

The coupled growth of population aging and international migration warrants attention on the methods and solutions available to adult children living overseas to provide distance caregiving for their aging parents. Despite living apart from their parents, the transnational informal care literature has indicated that first-generation immigrants remain committed to carry out their filial caregiving obligations in extensive and creative ways. With functions to remotely access health information enabled by emergency, wearable, motion, and video sensors, remote monitoring technologies (RMTs) may thus also allow these international migrants to be alerted in sudden changes and remain informed of their parent’s state of health. As technological solutions for caregiving, RMTs could allow independent living for older persons while any unusual deviations from normal health patterns are detected and appropriately supported. With a vignette of a distance care arrangement, we engage with concepts such as filial piety, in-absentia caregiving distress, and the social exchange theory, as well as the upholding of shifting cultural ideals to illustrate the complex dynamic of the satisfaction and quality of the informal caregiving relationship. This paper extends the traditional ethical issues in technology-aided caregiving, such as autonomy, privacy, and justice, to be considered within the context of distance care. We also posit newer ethical considerations such as consent in power imbalances, harm to caregivers, and stigma. These known and new ethical issues aim to encourage further ethically conscious design and use of RMTs to support distance care for older persons.

## Introduction

In light of global population aging, the efficient allocation and development of healthcare resources dedicated to the care of older persons have attracted wide international attention (Close et al. 2012; Comans et al. 2016; Lee et al. 2017; Patrão Neves, 2022; Petrou and Wolstenholme 2000; Safiliou-Rothschild 2009; Tang and Li 2022). It is becoming natural, in the global North, to live past 60 years of age and it remains a priority for older people and their families to ascertain that this longevity represents greater opportunities of health and happiness (WHO 2022). Not only has the number of 60 years of age has outnumbered children under ages of 5 in 2020, but the projected portion of those 60 years or older in 2030 would grow to become 1 in 6 of the world’s population. This age group would increase from 1 to 1.4 billion from 2020 to 2030, then doubling to 2.1 billion as projected in 2050.

Nevertheless, the aging process involves declines in functioning, increases in illnesses (e.g. osteoarthritis, pulmonary diseases, diabetes, etc.), higher risks of falls, and loneliness. These declines could result in reduced abilities to carry out both instrumental activities of daily living (IADLs) (e.g. care of others, care of pets, child rearing, communication, driving and mobility, financial management, home management, meal preparation, etc.) and basic activities of daily living (ADLs) (e.g. bathing, toileting, dressing, eating, feeding, functional mobility) (American Occupational Therapy Association 2020; WHO 2021). Many older adults now prefer to “age in place,” which is the ability to live comfortably and independently in a familiar environment while their functional and medical needs are supported (NIH National Institute on Aging 2017). The fields of medicine, health, technology, as well as the social sciences have gathered together in the past few decades to find new ways to support caregivers and older persons in fulfilling their desires to age comfortably. There are now technical developments within the fields of sensor technology, artificial intelligence (AI), and medical devices to enable an autonomous and independent transition into the later stages of life when more support becomes necessary (Liu et al. 2016; Majumder et al. 2017; Peetoom et al. 2015). By integrating ambient monitoring sensors within the home, medical emergencies adversely affecting older persons could be captured and reported to the desired caregiver, enabling continuous remote monitoring capacities and thereby reducing caregiving burdens for family members. On a broader industry level, this implementation of smart technologies in homes has been globally positively received. According to the Statista report in 2021, the revenue forecast for smart homes in the world increased from 39 billion US dollars in 2017 to 122 billion in 2022, and is predicted to continue increasing to 223 billion by 2027 (Statista, n.d.). The research community has also responded in turn, with several reviews investigating the empirical and ethical considerations relevant to these intelligent technologies for caregiving. Namely, a descriptive review published by Ienca et al. (2018a, b) found 539 intelligent assistive technologies for dementia, and a more recent systematic review by Felber et al. (2023) included 156 articles published from 2000 to 2020 on smart home health technologies for older care.

These innovative care solutions may also be instrumental in supporting older persons at a distance. International migration has risen steadily in the previous five decades, both in the number of persons and the proportion of the world’s population (McAuliffe and Triandafyllidou 2021). According to the World Migration Report, the estimated number of international migrants in 2020 is 281 million, or 3.6% of the global population, compared to 84 million and 2.3% in 1970. Under these transnational contexts, the ability of adult migrant children to carry out filial obligations would be impacted (Baldassar et al. 2006; Baldassar and Merla 2013). The physical barrier indicates limited accessibility to resources and social networks in the home country, administrative burdens from immigration policies, as well as additional difficulties navigating pertinent social security and healthcare systems (Tu 2023). These challenges, in turn, impact distance caregivers’ abilities to appropriately and consistently respond to the older persons’ varying needs. During exceptional periods, disruptions in political climates or the more recent COVID-19 pandemic only exacerbate these challenges in the distance care arrangement. Nevertheless, recent developments of diverse technologies are framed as new possible solutions to aid caregivers in providing emotional and psychological care from afar (Baldassar et al. 2016; Baldassar and Wilding 2019; Madianou 2014; Wilding and Baldassar 2018; Zechner 2008). Communication technologies such as unlimited international calls, social media platforms, and video calling software have created more ways to not only stay connected across a distance, but also to become more present with one another even when physically apart.

With the progressive development and mass production of smart technologies such as integrative sensors installed in the home, older persons could theoretically be supported using continuous monitoring of vital signs, daily routines, eating habits, and mobility patterns. When analyzed and presented through an interactive interface, older persons and their caregivers alike could stay connected remotely. Thus, smart technologies essentially reduce the need of caregiver to be in the same place with their older care recipient. These technologies enable caregivers to still provide support that could be handled at a distance, such as reminders for health check-ups, information related to improving health literacy, and emotional reassurance. In doing so, they are able to fulfill their filial duties as a child towards their aging parents.

In this paper, we will examine ethical issues that arise with the use of remote monitoring technologies (RMTs) in fulfilling filial care duties for the promotion of the health and wellbeing of older parents from a distance. The article will first introduce the current literature on transnational and distance care, which sets a foundation for examining the role and opportunities of the existing array RMTs. Next, we allude to the wealth of ethics research on the ethical concerns generally discussed when monitoring technology is used in the care for older persons. A case example of a distance care arrangement is then drawn to illustrate and highlight the need for additional deliberation when RMTs are used. We engaged the social exchange theory and cultural-specific filial obligations to understand the role of distance on autonomy, stigma, and quality of the caregiving relationship. In the filial care context, it may be beneficial to extend beyond the traditional scope of ethical considerations of older persons or caregivers as non-culture specific individuals, to evaluate the shared and relational well-being, as well as the satisfaction in the caregiving relationship of those involved as a familial unit in migration settings. To the best of our knowledge, the extension of ethical considerations in the use RMTs to caregiving at a distance has not been previously explored.

## What is Caregiving at a Distance?

Migration is one of the key challenges for family members in regards to providing proximate and in-person caregiving, prompting the development and codification of new and creative types of co-presence to maintain kinship and familial ties (Baldassar 2008). Being at a distance does not necessarily weaken responsibilities felt towards older parents. Though the caregiving responsibilities to parents and familial members are termed differently across various cultures, such as filial piety in Confucian cultures, filial devotion and love depicted towards older parents (as in the story of Shravan Kumar) in Indian cultures, and *Ketaatan Kepada Ibu Bapa* in the Malay culture, this paper uses the term “filial obligation” to encompass these senses of duty, responsibility, and respect that are morally tied to familial relations and previous sacrifices to provide both physical and emotional care for aging parents (Chang and Schneider 2010; Chappell and Funk 2011; Gui and Koropeckyj-Cox 2016; Li et al. 2021; Liu and Kendig 2000; Martani et al. 2021; Montayre et al. 2022; Schinkel 2012; Wangmo 2010). There are many studies that present how the responsibilities are managed by children living at distance and even divided among the children living close to the parent and at a distance (Cagle and Munn 2012; Chappell and Funk 2011; Kalavar et al. 2020; Wangmo 2010; Zechner 2008).

At the foreground of rising international migration, the research on transnational care also revealed complex dynamics of the legal statuses and reasons for migration (Sampaio and Carvalho 2022). Legal status and reasons for migration may inevitably influence the possibility to provide in-person care and perhaps the level of resources to entertain technological tools for caregiving. Economic migrants, especially those in middle-class transnational families, may have less legal concerns related to their ability to visit the home country and to explore costly technical tools that gives them greater options to enable their filial obligations (Moré 2022; Tu 2023). Furthermore, the number of siblings is another component that may affect the coordination of care activities and exchange of information to navigate healthcare and institutional resources, where research documented “local siblings” being granted respite from filial responsibilities when migrant children returns to visit (Kalavar et al. 2020; Kilkey and Merla 2014; Schroeder-Butterfill and Schonheinz 2019). On a similar note, the gendered aspect within caregiving has been widely cited and remains relevant in transnational caregiving, where emotional and hands-on components of care are viewed to be less challenging and are associated with daughters more than sons (De Silva 2018; Hequembourg and Brallier 2005; Russell 2007).

To conceptualize caregiving at a distance necessitates the definition of care in an established informal care framework. Distance could introduce a distinct set of challenges in financial, employment, health, political, and familial statues of caregivers, which may enable different care tasks and care provision consistencies. With the care ethics framework proposed by Fisher and Tronto (1990) and echoed by more contemporary researchers in transnational settings (Zechner 2008), caring is a process with four interrelated stages: caring about, taking care of, caregiving, and care-receiving. “caring about” involves simple connection through kinship, attention to needs, and affection, which coincides with the continuation of kinship in transnational families. This process involves the least commitment and resources from caregivers, but also includes frequent decision-making needs for caregivers especially when conflicting obligations arise. Thus, “taking care of” endows caregivers with greater power and responsibility as compared to the earlier stage. The subsequent “caregiving” stage engages with the more concrete “hands-on” tasks of care, with more intensive time and consistent commitments than all other stages (Fisher and Tronto 1990, 43). “Care-receiving” would be the responses of older parents towards distance care and the active participation to disclose information to the migrant children.

With the normalization of international migration, the normative conception of care limited to the “hands-on” caregiving tasks have been challenged to include other possibilities of a continuous multidirectional and asymmetrical circulation of care within the family (Baldassar 2016; Baldassar and Merla 2013). In this way, distance care tasks extend beyond this limited understanding to activities involved in “caring about” and “taking care of” such as the provision of emotional and spiritual care through intensive time and resource commitments from migrants living faraway. These are highlighted as “transnational social exchanges” that include the sharing of information, knowledge, health behaviors, and support for the promotion of health and well-being (Roosen et al. 2021, 2). Furthermore, technological developments have led to discussions of caregiving in the forms of ambient co-presence via polymedia environments or virtual co-presence through the exchanges of electronic messages (Baldassar 2015, 2016; Baldassar et al. 2006, 2016; Baldassar and Merla 2013).

For the purpose of this paper, the terms “distance caregiving,” “caregiving at a distance,” and “transnational caregiving” are used synonymously to fulfill the criteria of distance as a barrier in providing proximate care on demand. We note that in the field of gerontology, long-distance caregiving is typically defined as by caring for someone who lives at least one hour away, or broadly, any caregivers with complications due to the physical distance with the care recipient (Cagle and Munn 2012). There exists empirical studies investigating these challenges relevant to distance, in terms of time constraints and financial burdens to arrange visits, as well as psychological distress and anxiety, with the caregivers’ improvised solutions to bridge this distance with older persons (Edwards 2014; Sánchez 2022; Smith 2006; White et al. 2020). In this paper, we are focusing on the notion of distance care as a result of international migration, thus with greater caregiving barriers and even less physically accessible than compared with care provided across cities or regions within the same country.

## Remote Monitoring Technologies

As a way to ensure that older persons are aging safely and healthily at home, researchers are now looking at smart home technologies to allow caregivers to continuously monitor one’s physical, social, and cognitive conditions (Liu et al. 2016). The definitions of the “smart home” and monitoring technologies used for health are evolving as new technical capabilities emerge. One of the first definitions is by Aldrich (2003), whereby he defined a smart home as “a residence equipped with computing and information technology, which anticipates and responds to the needs of the occupants, working to promote their comfort, convenience, security, and entertainment through the management of technology within the home and connections to the world beyond” (Aldrich 2003, 17). A few years later, Demiris and Hensel (2008) defined the smart home as “a residence wired with technology features that monitor the well-being and activities of their residents to improve overall quality of life, increase independence, and prevent emergencies (Demiris and Hensel 2008, 33–34).” For the purpose of this paper, we focus on the smart sensor technologies that monitor and interact with each other to provide timely, specific, and health-related information to caregivers, rather than other smart assistive technologies to increase comfort, well-being, and efficiency of caregiving, such as vacuums, home appliances, and virtual reality devices (Wagner et al. 2012). With the help of a smart platform where signals and information are integrated, RMTs such as motion sensors, body-worn sensors, pressure sensors, video monitoring and sound recognition devices, detect and record activities of daily living, and significant health adverse events that occurs while the older person is at home (Peetoom et al. 2015; Wagner et al. 2012). Sensors could be either worn on the body, installed under the flooring, or as a part of carpeting to detect abnormal deviations in body postures and movements targeted towards the possible increased risk of falls and physical functional declines in the aging process (Wilson et al. 2015). Early detection of cognitive functional decline and wandering behaviors in dementia could also be monitored with the interoperable use of caregiving technologies in the home environment, while providing greater knowledge of daily habits and health data available for medical staff when necessary (Husebo et al. 2019). RMTs allow caregivers to check-in with older persons at any time both proactively or as a response to an alert, thereby designed to assist and partially replace the need for continuous human monitoring. With physical support being unavailable at a distance, RMTs could provide another way to maintain or enhance communication and kinship by creating virtual and ambient forms of contact that adds onto the existing methods of telecommunication via video calling and text exchanges (Benefield and Beck 2007; Bevan and Sparks 2011; Cagle and Munn 2012; Kalavar et al. 2020). RMTs also aid caregivers at a distance to partake first-hand in medical decision-making and the unearthing of potential health problems alongside the older persons (Benefield and Beck 2007; Mazanec et al. 2011). A qualitative study by Williamson et al. (2014) interviewed long-distance informal caregivers of older care-receivers, where all ten interviewed reacted positively towards the development of a mobile or website-based technology designated for long-distance caregivers with functions like video calling, medication, sleep, and cognitive health tracking, as well as reminder and asynchronous communication. It allowed distance caregivers to access more information on the daily lives of the older person and become more involved in their health, thereby alleviating the emotional burdens like feelings of guilt, stress, and worry. Key limitations mentioned by this study such as small sample sizes and inadequate explorations into privacy concern both indicate that additional empirical and normative research is due.

## Ethical Considerations related to the use of RMTs for Caregiving

Though smart technologies installed for the health and caregiving of older persons at home have been discussed to provide benefits for both older end-users and their caregivers, previous research has also strived to consider their design and use in terms of common ethical evaluations. In a descriptive review, Ienca and colleagues (2018b) examined ethical considerations critical in the design of intelligent assistive technologies for dementia care, and found that ethical issues fell into the following categories: autonomy, privacy, beneficence, non-maleficence, interdependence, and justice. Further literature has also brought forth additional ethical issues such as stigma, trust, and dignity (Birchley et al. 2017; Chung et al. 2016; Felber et al. 2022; McLean 2011). In the following paragraphs, we will delve a bit deeper into some of these ethical concerns.

### Autonomy

In the context of RMTs for caregiving, autonomy is related the ability to live and act in accordance with one’s own decisions, with independent living being one of its key components in caregiving for older persons (Ienca et al. 2018a, b; Varelius 2006). In face of declining health and independence, many older persons are not favorable towards moving into institutionalized care or arrange to co-reside with their caregivers in their homes (Mattimore et al. 1997; Roberto et al. 2001). When asked to appraise the installation of monitoring technologies in their places of residence, older adults expressed feeling greater independence from the functions to receiving reminders, health assistance, and emergency response services, which delayed the need for institutionalized care (Karlsen et al. 2019; Pais et al. 2020; Palm 2013). This finding is unsurprising, as one of the main tenets of RMTs developed for caregiving is to increase the ability to live independently at home (Lussier et al. 2020; Mazzu et al. 2008; Mehrabian et al. 2014). Nevertheless, it is critical to ascertain that the older persons under care are informed, provide, and continually reaffirm their consent for the use of monitoring in their care (Wangmo et al. 2019). The physical and temporal boundaries of technology use should also be delineated, in terms of the specific acceptable locations to install monitoring sensors and setting a schedule for their activation.

### Privacy

The current array of literature on privacy used in the frame of caregiving for older persons is multi-faceted and dynamic. Protections of the private sphere with the use of RMTs could be separated into three dimensions (Altman 1975; Burgoon 1982; Hughes 2004): (a) the physical, such as against the intrusion into one’s physical space with the installation of sensors (Ienca et al. 2021; Jaschinski et al. 2020); (b) the informational, sharing or lack thereof of information or data gathered from these devices (Ienca et al. 2021); and (c) the psychological, the threatening of one’s self-perception with access to “objective” sensor data incongruent with older persons’ memory (Parikh et al. 2015), or the automatic behavioral changes upon awareness of being monitored (Boissy et al. 2007; Cohen et al. 2016; Essén 2008; Jo et al. 2021).

In a relational caregiving context, privacy also allows older persons to place limits to their family or intimate partners from accessing their emotions, thoughts, and feelings, as well as the absence of undesired intrusions when making autonomous decisions (Mittelstadt et al. 2014; Schomakers and Ziefle 2019). In this way, protection to one’s privacy in turn facilitates autonomy and the freedom to live independently. When RMTs are used, the decisional privacy of older persons could be put at risk when they are interfered by the judgment and recommendations enabled by the analysis of health data collected. For example, data analysis could be carried out upon the older persons’ daily activity levels, food intake, or hygiene habits. Habit recommendations such as increasing movement, fluids, or designating certain times for sleep are generated that, in the best case, could promote health indicators. However, when scrutinized broadly and beyond the intention of these recommendations, they are at risk to influence, steer, or entirely eliminate the older person’s autonomy in the decision-making process.

### Benefit and No-Harm

Beneficence and non-maleficence work together as moral obligations to promote the welfare of, and avoid causing harm to, the older person (Ienca et al. 2018a, b). When discussed in the context of RMTs, it involves the ability to enhance well-being, social and mental states, or a healthier lifestyle and encouraging acts of self-management or self-care, and the prevention of health-averse behaviors and accidents. The use of sensors continuously monitors the cognitive, physical, and emotional states of the older person, and by comparing previously gathered data, identifies any deviations from daily living routines and reports these abnormalities timely to the caregivers or the older person themselves. By doing this, the use of RMTs could shorten the time needed to detect debilitating conditions, decreases risks of health-adverse situations, and increases feelings of reassurance. In addition, when the portfolio of RMTs include reminder management systems such as calendars, automatic pill dispensers, and digital interfaces, older persons could receive recommendations relevant to self-care or social activities, physical exercises, vital signs information, and reminders to take medication at regular intervals (Airola and Rasi 2020; Meiland et al. 2014).

### Interdependence

The relational aspect of interdependence is seen in the maintenance, promotion, and support of existing and new social relationships of the older person (Ienca et al. 2018a, b). In caregiving literature, it is often cited in terms of the effect of older age on loneliness and declining social inclusion, as well as the interactions between the caregiver and the care-receiver (Cohen-Mansfield and Perach 2015; Perissinotto et al. 2012). According to empirical literature that examines the role of technology use on older persons’ social interactions, the findings polarize into both improvements of the quality and quantity of social relationships, but also the fear of losing existing social bonds when technology is used as a substitute for in-person visitations (Bowes and McColgan 2013; Cahill et al. 2019; Chaumon et al. 2016; Essén, 2008; Kim and Chung 2015; Mazzu et al. 2008). Specifically, while RMTs could provide caregivers opportunities to check-in on older persons at any time and with reduced complications for travel (Bradford et al. 2018; Meiland et al. 2014), older persons express concerns that the wealth of information generated from RMTs may be enough to replace in-person visitations from their caregivers (Milligan et al. 2011; Pais et al. 2020).

### Justice

Issues of justice are discussed in terms of equal access, affordability, and availability of caregiving resources (Ho 2020; Ienca et al. 2018a, b; Jotterand et al. 2019; Vollmer Dahlke and Ory 2020). For technological solutions like RMT sensors, these involve the actual as well as the perception of cost, not only in terms of the one-off expenses, but also the efforts and price necessary for its maintenance and update to enable its continued use in the household (Hunter et al. 2020; Klemets et al. 2017). RMTs may involve few sensors, just as it may likely to be more evenly distributed in every space of the home with multiple functionalities, which may cause a technological divide within the generation of older persons who are open to accepting RMTs.

## Caregiving at the Distance Using RMTs: Extending Ethical Considerations

In this section, the commonly discussed ethical issues in proximate caregiving literature will be extended and highlighted with particular dimensions that become relevant for care at a distance. We will use a vignette to tease out new ethical concerns that arise when immigrant children use RMTs to care for an older parent. The following caregiving arrangement is inspired by a qualitative interview with an international immigrant providing care at a distance to an older parent.

*Mrs. Yana is 45 years old and lives with her spouse and 2 children in Switzerland. She is the only child of her father, Mr. Zan, 77 years old, who lives in their home country in another continent. Mr. Zan has been living alone since his wife passed away 5 years ago. Due to declines in his mobility and physical functioning, he now requires assistance for IADLs and ADLs from professional aids that provide home-visits twice a day. While Mr. Zan has occasional social activities as part of the neighborhood outreach program, he does not have immediate family or relatives nearby. Because Mrs. Yana has to care for her school-age children, she can only visit her father once a year. Apart from these visits, a schedule is set up for audio or video calling each week so that any health updates can be shared with the daughter, and to converse about each other’s thoughts and day-to-day challenges.*

*Mrs. Yana works as a technology consultant. Due to her knowledge and experience with health technology, she gifted her father around 2 years ago a wearable device to monitor his blood oxygen levels, heart rate, and sleeping schedules. With the consent of her father, his health data are shared on her smart phone in real-time, which provides alerts when there is an unusual pattern in her father’s routine or health. She has also programmed the wearable device to alert emergency services located near her father in face of dire health changes and regularly shares the available health data to a medical professional to monitor for abnormal patterns. At the same time, Mrs. Yana can provide emotional reassurance, technical troubleshooting including device updates, as well as maintain an additional communication channel with her father to adjust for challenges in time-difference and flexibility from afar.*

*Two weeks ago, Mr. Zan took a bad fall. His abnormal posture and sudden change in velocity activated the fall sensor on his wearable device, which automatically called the emergency services in his city. He was then taken to the hospital in an ambulance and accompanied by his neighbors’ son, who was staying with his parents for the weekend.*

*Since this incident, both father and daughter have been in discussion about installing smart monitoring technologies to provide greater supervision and immediate support should there be a need. These discussions begin with Mr. Zan refusing to transition to institutionalized care or group homes, and wanting to remain in a familiar environment. In light of his increasing frailty, he has recently agreed to install RMTs with ambient and video sensors in discreet areas of his home at the request of his daughter. It now allows her to continuously monitor his health and turn on the video whenever necessary.*

*This afternoon, Mrs. Yana is alerted to her RMT interface where her father seemed to be suffering from a cardiac event and has taken to the ground unconscious. She calls the ambulance immediately and watches on her interface while waiting anxiously for help to arrive…*

The above vignette describes the reality of many distance caregivers which involves simultaneously balancing many conflicting obligations for work, their own nuclear family in the host country, and the increasing filial obligations with the shifting health demands of aging parents back home. Being the primary informal caregiver and the only child of Mr. Zan, Mrs. Yana seems to be dedicating intensive time-commitments to provide consistent emotional care from afar, practical support for technologies, and liaison with medical professionals for health-specific challenges, as well as taking responsibility to react appropriately to her father’s needs. In this way, though she is not physically present and is not a medically trained professional, she still provides both resources for medical care and care in a wider social-human sense through virtual forms of co-presence. Below, we tease out several ethical concerns that arise when RMTs are used in such a caregiving context delineated in this vignette.

### Autonomy and Distance Caregiving

The traditional principle of autonomy is related to the freedom for self-governance and living a life that is authentic to the individual (Beauchamp and Childress 2019; Varelius 2006). To incorporate this in the caregiving relationship of older persons with a distance caregiver using RMTs, it becomes important to examine the older persons’ values on independence in the context of the caregiving relationship.

The social exchange theory asserts that the quality and inherent satisfaction of the intergenerational relationship could be contingent on the relative balance of power between an older person and their family caregivers (Brackbill and Kitch 1991). This facilitative power is defined by the level of dependency on and the provision of exchangeable resources to one another. The relationship may improve with a more equal distribution of power, and worsen when one has a power advantage over another. In the care context, the older person would have more facilitative power when there is greater overall independence and from their caregivers (Schicktanz and Schweda 2021). Conversely, an increased dependence on caregivers may convey a loss of facilitative power and a negative impact on the caregiving relationship.

Though this social exchange theory was developed from co-residence or proximate care arrangements, it can also be extended to distance care and technologies. Interpersonal conflicts between the informal caregivers and older persons may also arise from the use of RMTs, when such use is not in accordance with the older persons’ wishes and sense of territoriality (Berridge et al. 2020). Often as surrogate decision-makers, family caregivers are cited to place greater value on aspects of safety, efficiency, and health benefits of their care receivers than the maintenance of their autonomy (Shelton et al. 2018). When relieving caregiving burden is also a benefit from such monitoring (Ienca et al. 2018a, b; Robinson et al. 2020), it becomes of greater importance to ensure that such devises are used in accordance with the wishes of older person and not the wishes of the caregiver solely. The older persons’ abilities to decline or modify their desires to accept recommendations from informal caregivers in regard to RMTs need to be considered (Berridge et al. 2022). This issue could be particularly relevant when the opinions of family caregivers living at a distance are greatly valued by their older parents. As a common exchange of caregiving in transnational care, financial remittances sent to the parents could also create imbalances in power (Laidlaw et al. 2010; Lan 2002; Treas and Mazumdar 2002, 2004; Zhou 2012).

In the case example above, the immigrant daughter may have greater power as the one who recommends or gifts the technology to her father. With her knowledge of existing technologies and perhaps even greater purchasing power, the power balance is further tilted on her side. In these cases, more attention is needed to ensure that older parents autonomously supports the use and continues to agree to use RMTs (Zhu et al. 2021). It is thus imperative that children also take into account any biases, prior knowledge, and invested interests in their technological recommendation that could influence their parents in exercising their decision-making capacity. Despite consent procedures (particularly in the written form) for legal purposes often present in more professional caregiving settings and are not commonly stipulated in family caregiving contexts, there remains the necessity to obtain consent (appropriate for the regional and familial context whether its oral or implied) from the older parent to use technologies, to be monitored, and for personal data to be shared, in the context of fulfilling filial duties at a distance. Reaffirming or “rolling informed consent” ensures any changes in opinion of the older parents could be taken into account, especially when there are cognitive deteriorating conditions that may affect the expectations and understandings of data sharing (Novitzky et al. 2015). In the context of family caregiving and persons with dementia, this kind of a consent procedure becomes a communicative process and considers the changing competencies of the older persons. In addition, with respect to older persons and caregivers in different societies, we acknowledge that it remains important to consider the aspect of relational autonomy and the balancing of other ethical issues (i.e., responsibility to provide care to older parents, trust between the parent and the child) at play, rather than generalizing the consent procedure to written consent and liability procedure as common in certain cultural contexts across all family caregiving arrangements.

On the other hand, once the installation of RMTs is completed in accordance to the maximization of autonomy and comfort of older persons, they may improve the caregiving relationship between the older person and their distance caregivers. As a rule, the enhancement or interference of autonomy with the use of RMTs at a distance could be seen as linked to the power dynamic in the care relationship. Empowerment of the older parents and an improvement of the care relationship may be more likely to occur if and when the devices are purchased or used autonomously. However, if the children bought it for older people and there may be a difference of knowledge or desire, one should be cautious to avoid a state where the older person’s autonomy could be harmed due to increased power imbalances in the care relationship.

### Privacy and Caregiving at a Distance

When RMTs are used for caregiving at a distance, we are faced with the question: “What kinds of decisions or situations would it be unethical or potentially dangerous for RMTs to be involved in, when children are caring at a distance?” As elaborated in earlier sections, RMTs may provide feedback from accumulated data analysis of daily routines to older persons, such as altering diets or exercise to fulfill a certain health-related goal. In the distance care cases, when close family members, especially primary caregivers, are located overseas, it is challenging to access the status and health of older persons in a timely and holistic manner. Drawing back to the concept of decisional or psychological privacy as a barrier to unwanted influences when making decisions, these suggestions could add and thus alter the array of information presented to older persons when making daily health decisions, even steering them towards a certain direction to carry out activities to fulfill a health goal. Any such interference or steering is therefore an external source of manipulation and a violation to one’s privacy. In the current technological maturity or future possible unreliability of health-related recommendations from RMTs, habit-altering recommendations could also be unwelcome (Rosman et al. 2020). With distance, informal caregivers need to both overcome significant obstacles to access enough information about the older person under care and pose timely interferences to counter any negative consequences derived from these technologies. Therefore, it is important that caregivers at a distance do not depend solely on the monitoring information. They also need to ascertain that the RMTs are not generating automatic feedback recommendations that could be harmful to the psychological privacy of older persons. Though this problem persists in proximate caregiving, it is particularly problematic when distance caregivers cannot access the same dimension of information as when they are close by nor assess subtle environmental cues that indicate something is amiss (Mahoney 2011).

What if we suppose that distance informal caregivers will not be given permission from older persons to access the health data from RMTs? While RMTs could be set up with the assistance of the caregiver, older persons should still be able to exercise their decisional autonomy and decide whether their movements, habits, health data, and alerts be sent to caregivers at a distance. There have been extensive calls to ensure the ability of end-users to control technology when needed (Epstein et al. 2016; Londei et al. 2009; Van Berlo 2011). Here, the question becomes: “What kinds of data would older parents not want to share with their children living at a distance?” On the one hand, older parents such as Mr. Zan may find it embarrassing to share intimate details with their habits and daily routines with his daughter. In such cases, they must have the ability to control such information flow. Also, older parents may not want their children to be alerted by information that could be morally stressful, in emergency situations where the latter could not provide help due to distance (Mazanec et al. 2011). In the literature on communication and emotional support in transnational care, careful monitoring of information shared and withholding negative experiences, particularly bad news and truths about their health and well-being, are rationalized with protecting each other from pain and worry (Baldassar 2007, 2015). For example, when asked about the reasons to withhold news relevant to a serious illness diagnosis in the family, there were allusions to the inability to react “anyway living so far away” and plans to deliver this news in the next in-person visit (Baldassar 2007, 402). As the goal of the RMTs is to monitor for events that could possibly harm the older person, careful discussion between the caregivers and care-receivers are necessary to limit invasion into the private life of the older persons. Doing so not only ensures the safety of the older parent, but also allows the child to fulfil the responsibilities they feel.

### Stigmatization and Filial Piety

Care provision to older parents is often viewed as a responsibility, if not a generational contract, or expectation (Baldassar and Merla 2013). It adheres to the corresponding social norm of the community as a way to ensure the survival of kin and family. A qualitative study by Gui and Koropeckyj-Cox (2016) in Canada highlighted the dilemmas faced by Chinese only children, as they attempt to negotiate their filial obligations to parents living at a distance. Even while these interviewees may be receiving education and influences from a culture that values individualism and independence, their parents upholds caregiving values of the home country that includes “responsibility, sacrifice, and collectivism” (Gui and Koropeckyj-Cox 2016, 268). In Chinese cultures, for example, where filial behaviors to provide care and respect to parents are rewarded and highly valued as virtuous and indispensable obligations that give back to the care given by parent to young children. Associated with the filial expectation is a stronger emphasis on the commitment to be present in times of stress and perform hands-on caregiving for older parents. The absence of this care can thus be labeled as unvirtuous and ungrateful. Another example is the reluctance to take care of family members in the home and send them off to nursing homes, which can be seen as morally wrong or at odds with the filial expectation (Gui and Koropeckyj-Cox 2016; Kao and McHugh 2004; Tinker 2001). As the lives of families in collectivist cultures are closely intertwined, this could lead the surrounding community to stigmatize both the child and the parents involved as a “bad example” of a virtuous and dutiful family.

Similarly, the lack of hands-on care can also be extended to the distance care arrangement, where any signs of prolonged absence or inability to fulfill caregiving responsibilities that are at odds with the social ideal can lead to stigmatization from those in the immediate social community. If RMTs could begin to be used to enable virtual co-presence, more consideration is needed towards the potential stigmatization towards older parents. More specifically, in the distance care arrangement, gifting the technology may highlight the barrier to provide proximate or hands-on care to surrounding community. In a typology of transnational arrangements, Kilkey and Merla (2014) identified the “remainers” as those distance caregivers and care-receivers who are separated by geographical distance and are relatively immobile. Different from other types of transnational carers, they neither provide care during short in-person visits nor plan to repatriate to the home country for caregiving. In this way, the distance care arrangement may proceed to continue for the long-term and would most likely be required to consider technological tools such as RMTs. In this way, one could subsequently associate the use of RMTs with the long-term arrangement for “remainers” that is at odds with cultural expectations for caregiving. Especially for more expensive RMTs or devices that require more initial costs to set up and would be a greater investment for caregivers, this would consequently be associated with the expectation that the technology could be used for a longer period of time in which the caregiver is physically absent. On the contrary, if caregivers expect and are making plans to repatriate, perhaps the technology would not be immediately needed. Using the vignette provided as a case in point, there is a difference between Mrs. Yana initially gifting the wearable device to her father compared to the installation of a more complex remote monitoring system. The issue of stigma extends beyond the judgment or reason within the caregiving relationship and externally towards the views of those in the community. In other words, apart from obtaining greater details on Mr. Zan’s health condition and reason for his persistent falls, the investment of RMTs may signal to others than the distance care arrangement may continue instead of Mrs. Yana’s immediate repatriation to care for her father.

Nevertheless, the traditional value of filial piety does not limit itself to caring by one’s side, but also includes other kinds of care like social contact, establishing good social contact with the parent’s social community to care in emergencies, or to find a compatible caregiver for the parents (Gui and Koropeckyj-Cox 2016). Therefore, the use, and especially gift, of RMTs could also symbolize positive financial status of the children, a sign that the child is doing well for themselves aboard and would like to bear the costs of improving solutions for communication with their parents.

### Harm to the Caregiver

A wealth of research on the mirroring effect of older persons’ suffering on their caregivers, causing emotional and physical distress to the caregivers (Brody 1985; Capistrant 2016; Cuijpers 2005; Monin and Schulz 2009; Schulz et al. 2020). When parents have underlying health issues such as unpredictable and uncontrollable emergency events (e.g., risk of falls, psychological instability), family caregivers may incur psychological stress from constantly bearing high levels of vigilance and preparations to adapt to new caring responsibilities (Schulz et al. 2020). However, the ability for subsequent medical rescue and treatment depends on various additional factors, such as the timeliness of emergency services and social or community assistance. In the context of transnational and distance caregiving, researchers Kalavar and colleagues (2020) used the term “in-absentia caregiver stress” to indicate that distance caregivers may experience anxiety, guilt, worry, shame, helplessness, and stress, sometimes greater than that felt by proximate caregivers. This feeling of guilt and helplessness from the failure to provide in-person support is also echoed in other articles (Baldassar et al. 2006; Cagle and Munn 2012). One of the main sources of concern and psychological stress for caregivers is during emergency situations when they are not able to be physically present (Kalavar et al. 2020), as alluded to in the case study.

That is, the case of Mrs. Yana and Mr. Zan provides context to challenge the “do-not-harm” principle, as seen in the helplessness that is particular to caregivers with high barriers to provide physical support. The questions with the use of RMTs would now become: if the ambulance does not arrive timely and her father has unfortunately passed away despite the timely call for emergency rescue, what are the psychological implications for Mrs. Yana, who, by virtue of the RMTs, now not only needs to reconcile with the in-absentia caregiver stress, but has to bear witness to the entire process? How could the caregivers’ emotional impact in such emergencies be best ethically evaluated, when they are not only unable to help but now have visual data to supplement this experience?

While the answers to these questions are inherently individual, we would like to approach this issue with an extension of the existing literature on guilt as a motivator in transnational care. According to Baldassar (2015), moving away from kin inherently causes the migrant child to be left with difficult moral decisions in regards to planning for the care of aging parents. This physical absence is at odds with the normalized concept of caregiving being hands-on and within proximate contexts. When migrants are unable to close this distance with the older person, guilt may motivate them to use all methods available to maintain communication and create opportunities to care in other ways, such as through virtual co-presence. It becomes easy to imagine the possible reception towards the use of RMTs to care for kin located across far distances, as an extension of this motivation to stay in touch and continue care provision.

The concept of guilt as a motivating theory may help to unpack the increased harm to distance caregivers as compared to those located in proximity. For example, compared to caregivers located in the same city, but living on a different street, community, or even temporarily away, distance caregivers are particularly susceptible to guilt and will therefore be more impacted by the availability of monitoring data in emergencies. It reminds them that it is not possible to be there, that care is limited and has always been contrary to the ideal of caregiving being hands-on. The availability of this footage therefore may harm distance caregivers as it is a constant reminder of their decision to create distance between themselves and their kin. It is for this reason that distance caregivers should take this possible increased harm into consideration when planning to employ RMTs, as additional technologies to enable virtual co-presence may also mean increasing psychological harm.

## Limitations

In this manuscript, we did not ponder on the legal concerns such as how consent should be specifically sought in light of the international distance as well as different ways of consenting in different cultures. Also, issues related to data ownership and data protection are not included. Due to the nature of transnational caregiving and the use of RMTs, there is an increasing need for critical research into the relevant legal entities responsible for the data of older persons, consent procedures between data sharing with children, and more broadly, the data protection regulations in various countries and from different manufacturers of technological devices. Our analysis is based on a case example of economic migrant who is the only child. Migrants with varying levels of financial comfort, resources to share filial duties, or has traveled internationally for political reasons would nevertheless have different levels of motivation to allow collection and sharing of intimate health data of their parents (Sampaio and Carvalho 2022). For political immigrants or those with particular reservations towards surveillance and data security, there may be legal or political reasons that extend beyond ethical concerns to forgo RMTs for caregiving and health purposes. Hence, it remains to be seen whether and to what extend political situation extent the analysis that we have carried out in this paper, mainly for economic migrant’s filial obligations.

Previously in the paper, we elaborated that having siblings has multi-fold effects, both on the increase of filial resources and the exacerbation of gender roles in caregiving. Along this line of sharing care responsibilities is also the issue of the greater social networks (extended family, friends, community, and the local system) of the older parent. Specifically, power and influence could originate from a source other than Mrs. Yana, such as other relatives of her father, advice from technology companies, relevant policy guidance in both countries, as well as the personalized feedback from Mr. Zan’s professional caregivers. Though these warrant their own discussion in another publication focused more broadly on the influence of social factors in accepting or rejecting technology, they should remain part of the ethical consideration to ensure that Mr. Zan’s use of technology is autonomous and would thus lead to positive health benefits.

Finally, though this paper focuses on health-related needs and challenges from the perspective of using RMTs for caregiving towards health promotion purposes, reality nevertheless involves further administrative and bureaucratic tasks that are often left with distance caregivers to resolve from afar. These and other challenges should be of greater focus in upcoming empirical papers to allow a more holistic understanding and subsequent support for these caregivers of older persons.

## Conclusions

As care is typically seen as being hands-on and providing practical support, caregiving at a distance poses obvious challenges to ideally fulfill older persons’ needs at home. When the caregiver is unable to immediately respond to calls for help, to change and replace household items on demand for older persons, or even to experience these challenges first-hand, the caregiving responsibilities also transforms at a distance. Using RMTs to send and receive timely alerts for help or to fulfill social connectedness needs, the distance caregiver is now able to fulfill greater caregiving tasks previously not done. Under these contexts, RMTs could not only provide health information to caregivers, expand current channels of communication, but also enable a virtual form of co-presence. These prompt further investigation into their possible role to improve care solutions available for international migrants with caregiving responsibilities to older parents back home. Whereas depictions of caregiving technologies often elicit hints of increased loneliness or fears of human replacement in proximate care, technology use may provide the “second-best” option for distance caregivers when the barriers to physically care or visit are higher and care is provided in other forms like remittance or telecommunication (Barnier and Chekkar 2018; Cahill et al. 2019; Chaumon et al. 2016). In the existing literature on transnational care, researchers note that additional forms of co-presence could also be experienced and appreciated by families, such as “virtual” and “ambient” forms of co-presence (Baldassar 2008; Madianou 2016). In an empirical study, Cabalquinto (2018) investigated the distance care provided by Filipino adult migrants living in Australia who used mobile devices to foster a type of co-presence and “togetherness” across the distance. These acts are motivated by the need to maintain family values and exercising filial obligations that are made more tangible and carried in a vestibule through the use of new communication technologies. Cabalquinto (2018) argues that care initiates from the moment that the device is sent, when needed, to the parents in the home country for the purpose of maintaining contact across the distance. Care is continued by the purchase of an appropriate phone plan or finding ways to ensure constant connection via the device. Extending this to the use of RMTs, the acts of suggesting, purchasing, setting up, and making preparations for troubleshooting are all ways that caregivers like Mrs. Yana could improve care and social connection in the existing distance care arrangement for her father.

Nevertheless, there are new challenges that those involved must heed attention to, such as the potential invasiveness to private spheres, the effect of power imbalances on autonomy, the potential exacerbation of loneliness and stigmatization towards older persons, as well as the harms to caregivers. The latter two may not be previously anticipated risks. With the use of RMTs, there were also interactions between the ethical considerations. Namely, older persons were willing to trade-off a certain amount of privacy to increase independence, allowing their caregivers to view their data through activity monitoring technology as a way to prolong aging in place (Essén 2008; Mahoney 2011; Townsend et al. 2011). In addition, researchers caution that informal caregivers shall not be delegated additional rights to interfere or disrupt the autonomy of older persons by virtue of purchasing the technology (Mitseva et al. 2012). At the same time, responsibilities of the distance caregiver can increase with the additional obligation to refer and check more frequently on the status of the older person. This increase to caregiving burdens could also be subject to greater consideration. Though the discussion of these ethical issues should not discourage end-users from the use of RMTs for caregiving at a distance, their elaboration in this paper could support and allow end-users to adequately weigh their opportunities against their risks. The ethical trade-off between the benefits of RMTs must be considered with these additional ethical considerations prior to their introduction to distance caregiving and urges the research community to build further upon them.

## Data Availability

Not applicable.
